# Metasurface eyepiece for augmented reality

**DOI:** 10.1038/s41467-018-07011-5

**Published:** 2018-11-01

**Authors:** Gun-Yeal Lee, Jong-Young Hong, SoonHyoung Hwang, Seokil Moon, Hyeokjung Kang, Sohee Jeon, Hwi Kim, Jun-Ho Jeong, Byoungho Lee

**Affiliations:** 1 School of Electrical and Computer Engineering, Seoul National University, 1 Gwanak-ro, Gwanak-gu, Seoul, 08826 South Korea; 2 Nano-Convergence Mechanical Systems Research Division, Korea Institute of Machinery and Materials, 156 Gajeongbuk-ro, Youseong-gu, Daejeon, 34103 South Korea; 3 Department of Electronics and Information Engineering, Korea University, 2511 Sejong-ro, Sejong, 30019 South Korea

## Abstract

Recently, metasurfaces composed of artificially fabricated subwavelength structures have shown remarkable potential for the manipulation of light with unprecedented functionality. Here, we first demonstrate a metasurface application to realize a compact near-eye display system for augmented reality with a wide field of view. A key component is a see-through metalens with an anisotropic response, a high numerical aperture with a large aperture, and broadband characteristics. By virtue of these high-performance features, the metalens can overcome the existing bottleneck imposed by the narrow field of view and bulkiness of current systems, which hinders their usability and further development. Experimental demonstrations with a nanoimprinted large-area see-through metalens are reported, showing full-color imaging with a wide field of view and feasibility of mass production. This work on novel metasurface applications shows great potential for the development of optical display systems for future consumer electronics and computer vision applications.

## Abstract (web summary)

Here, the authors demonstrate a metasurface application to realize a compact near-eye display system for augmented reality with a wide field of view, full-color imaging, high resolution and a sufficiently large eyebox.

## Introduction

Metasurfaces are planar optical elements composed of artificially fabricated subwavelength structures, and they have attracted considerable interest owing to their powerful and versatile performance in modifying electromagnetic characteristics^1–5^. Recent advances in metasurfaces show that they can overcome the limitations of conventional bulky optical components, which have restricted further development of miniature optical and electronic devices^6–12^. Among the various types of metasurfaces, metasurface lenses, also called metalenses, are regarded as promising metasurface platforms with great potential for practical application. Thus, pioneering works addressing various concepts related to metalenses have been reported in recent years. For instance, dielectric metasurfaces composed of silicon posts enable the realization of metalenses with high numerical aperture (NA) and polarization-selective multi-functional metasurfaces under linearly polarized light^6,13,14^. Metalenses based on the spin-rotation coupling of light also exhibit remarkable performance with broadband characteristics^15–18^. Thanks to the high performance and compactness of such metalenses, very recent progress in the field of metasurfaces has revealed the potential of metalenses for use in future optical devices.

Augmented reality (AR) is a technology that integrates computer-generated virtual information into the real world and is regarded as the next generation of display technology. For the realization of AR, various technologies have been developed to reduce the sense of heterogeneity between the virtual information and the real-world scene to provide the user with an immersive experience^19–40^. Among the various approaches, see-through near-eye displays called glasses for augmented reality (AR glasses) have been receiving the greatest attention because of their potential to provide an extremely high sense of immersion. In AR glasses, a display placed in front of a human eye produces a virtual image and allows the user to naturally experience a mixture of virtual information and the real world. Numerous studies addressing the challenges of such platforms have been reported, and several commercial products have been launched^27–40^. To realize a see-through near-eye display, a transparent eyepiece is required, which must be able to float a generated image to the desired location in the real-world scene. To this end, various eyepieces have been proposed, such as those based on spherical mirrors, free-form optics, diffractive optical elements (DOEs), and holographic optical elements (HOEs)^29–39^. They are motivated by the concept of a see-through near-eye display realized on the basis of their own optical properties, but due to the limitations of conventional optics, there remain several challenges to realize high usability and true mobility of AR glasses. In particular, for the realization of ultimate AR glasses, it is necessary to provide an ultra-wide field of view (FOV), a compact form factor and a sufficient eyebox in which the intact image can be observed in the pupil, but no clear solution to all of these problems has yet emerged.

Here, we propose a metasurface application that enables an AR near-eye display with an ultra-wide FOV, full-color imaging, high resolution and a sufficiently large eyebox, which has not yet been reported. To this end, a see-through metalens with a high NA, a large-area, broadband characteristics, and an engineered anisotropic optical response is proposed. By virtue of the anisotropic optical response, the see-through metalens can perform two different optical functions: it can serve as an imaging lens for virtual information and as transparent glass through which to view a real-world scene. Since these two optical functions can be provided at the same time, the see-through metalens can be positioned right in front of the eye without any optical components like conventional glasses. Thus, it has a much wider FOV than conventional see-through near-eye displays under the same conditions. To fully utilize this anisotropic optical response, our metalens has been customized to satisfy the criteria for AR applications. We have implemented a see-through near-eye display with the see-through metalens with high transmission and enough modulation efficiency throughout the entire visible region. We have fabricated a large-area see-through metalens with a high NA (diameter of 20 mm and NA of 0.61) based on the nanoimprint technology, showing feasibility of the metalens for practical mass-production. Based on this, we experimentally realized a wide FOV (90°) in our prototype display system. In addition, by increasing the lens diameter to 35 mm, the FOV can be increased to more than 120°, which has been regarded as an impossible area in conventional AR display. This work is expected to be a significant advance in many areas including wearable devices, future optical displays, computer vision, wearable electronics, biological imaging, medical devices, and optical microscopy. Moreover, it is worth noting that this research demonstrates the great potential of metasurfaces for practical application in our daily lives.

## Results

### Augmented reality near-eye display with a see-through metalens

**Fig. 1:**
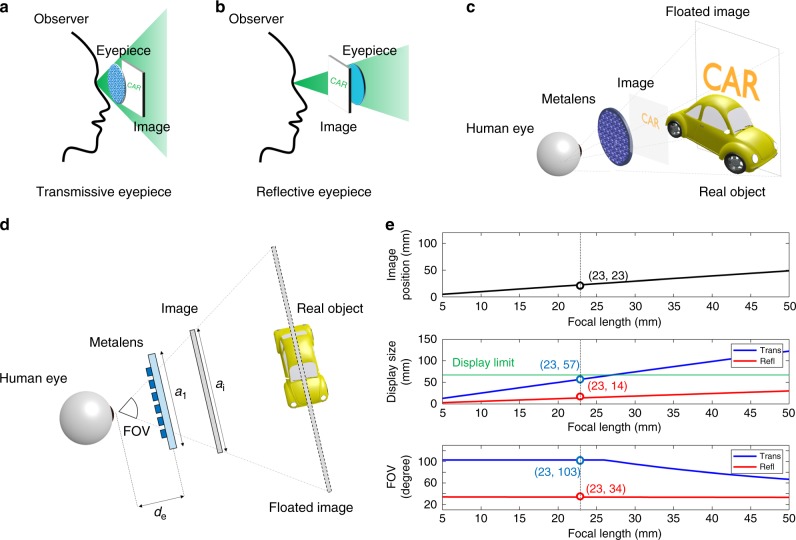
General illustration of the transmission-type eyepiece for augmented reality. **a**, **b** Concept illustrations of transmissive eyepiece and reflective eyepiece. **c** Schematic illustration of the see-through near-eye display system with a metalens as a transmission-type eyepiece. **d** Configuration of the system with the detailed parameters. **e** Specifications and FOV of the proposed system. The green line notated as display limit represents the maximum display size in which two displays in front of both eyes can exist without physically interfering with each other

In conventional AR glasses, light from a real-world scene passes through transparent optical elements such as half spherical mirrors, DOEs, and HOEs. Meanwhile, virtual images from a source such as a laser projector or a display panel are reflected or diffracted by the optical elements and imaged onto the user’s eye. Finally, the real-world scene and the virtual images are integrated into the human eye. See-through near-eye displays with conventional optics are inherently hindered by the low performance characteristics of the conventional optical elements, with the low NA and high thickness inducing a narrow FOV and a large form factor. Above all, such displays use a reflective optical element to float virtual information to a desired depth, thereby limiting the ability to extend the FOV^29–39^. They require space for transmitting the image to the eyepiece; consequently, the eye relief which is the distance between the eyepiece and the human eye is inevitably increased to produce a larger image as shown in the Fig. 1a.

This problem can be solved by placing the eyepiece right in front of the user’s eyes as shown in Fig. 1b. If the lens is positioned right in front of the eye, the FOV of the system will be determined solely by the eye relief and the lens aperture. In other words, there is no correlation between the display size and the eye relief distance, so the display size can be enlarged to an unlimited extent. Thus, the FOV can be enlarged without restriction by simply increasing the lens aperture. This is the advantage of a transmission-type eyepiece compared with conventional eyepieces. Although other approaches for placing the imaging lenses in front of the eye have been suggested^40^, no clear method of solving this issue has yet emerged due to the limitations of current photonic technology. Therefore, we propose a see-through metalens with performance beyond that of current conventional photonic technology for use as a transmission-type eyepiece. This see-through metalens can selectively act as a lens for virtual imaging and as transparent glass through which to view a real-world scene when the device is located in front of the eye.

Figure 1c shows a general illustration of the proposed system using the see-through metalens. As shown in the Fig. 1c, the see-through metalens is placed in front of the human eye, and a transparent image is placed within the focal length of the lens. The transparent image can be directly implemented using a transparent screen or can be transmitted by existing optical elements such as diffuser HOE, scattering polarizer, beam splitters, or lightguide.

The transparent image is floated to the desired location in the real-world scene by the see-through metalens, while the light from the real-world scene enters the human eye without distortion. This is the general concept of a transmission-type eyepiece that is used in the proposed see-through near-eye display. Figure 1d shows the configuration of the proposed system with an explanation of the detailed parameters. The maximum FOV is expressed by a simple relation between the lens aperture and the eye relief distance: FOV = 2tan^−1^(*a*_l_/2*d*_e_), where *a*_l_ is the lens aperture and *d*_e_ is the eye relief distance. Meanwhile, the required display size (*a*_i_) for the maximum FOV is expressed as *fa*_l_/*d*_e_, where *f* is the focal length of the metalens. For simplicity of explanation, suppose that the image is floated to infinity and that the eyebox is fixed. The focal length of the metalens determines the required display size, which is related to the form factor of the system. Figure 1e shows the relationship between the required form factor and FOV of the proposed system. The image position indicates the distance between the image and the metalens, the display size is required display size for providing maximum FOV, and the FOV is maximum FOV. This simulation is conducted under the similar condition of commercial products where the lens aperture is 35 mm, floating depth is 2000 mm, eyebox is 10 mm, and eye relief is 10 mm (see the Supplementary Note 1 and Supplementary Figs. 1 and 2 for detailed simulation condition and method). For a transmission-type eyepiece, the proposed system can have an ultra-wide field of view of more than 100° with a 10 mm eyebox. Meanwhile, the required display size and the image position are proportional to the focal length. Therefore, the transmission-type eyepiece should have a short focal length and a large aperture to simultaneously increase a compactness of the system and a wide FOV. In other words, a large NA is required for this transmission-type eyepiece. In this respect, a see-through metalens with a large NA and large area guarantees a compact eyepiece with a wide FOV, which is why we have chosen such a metasurface as the eyepiece for our next-generation AR head-mounted display (HMD).

### A see-through metalens: a novel eyepiece for augmented reality

**Fig. 2:**
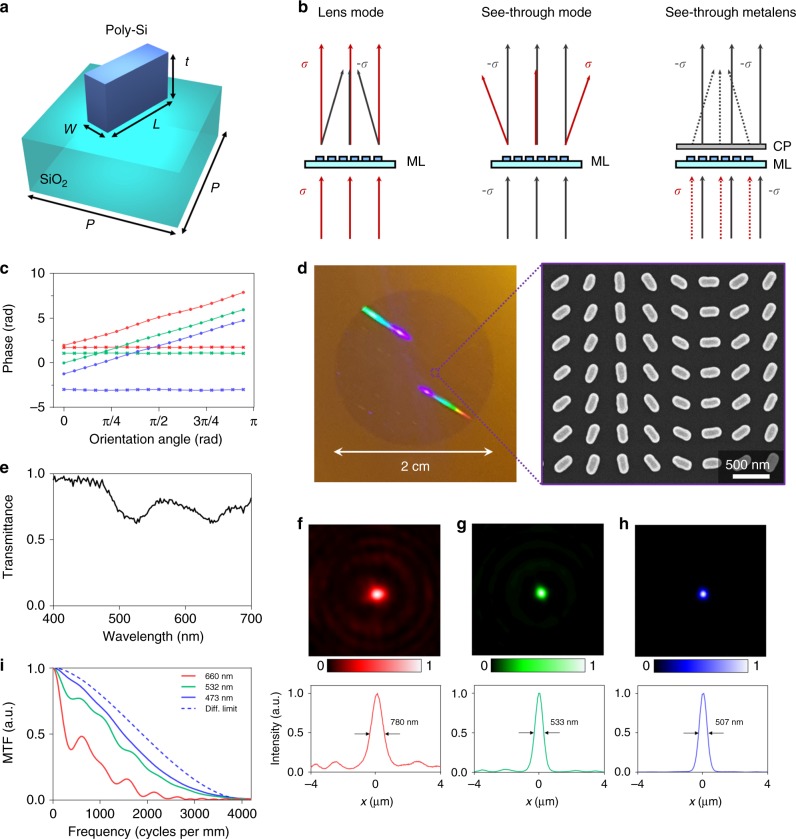
Operating mechanisms and demonstration of a see-through metalens. **a** Schematic illustration of a unit cell of the see-through metalens. The meta-atom is characterized by several parameters, such as the length *L*, the width *w*, the period *P* and the thickness *t*. **b** Schematic illustrations of the optical behavior of the see-through metalens under several incident light conditions. ML and CP denote the metalens and a circular polarizer, respectively. **c** Plot of the phase as a function of the nanorod orientation angle. Red, green, and blue lines are for the wavelengths of 660, 532, and 473 nm, respectively. Cross marks are for co-polarization while circle marks are for cross-polarization. **d** Photograph of the fabricated see-through metalens with a diameter of 20 mm. As seen, this device has the property of transparency. An SEM image of part of the device is shown in the inset. The scale bar represents 500 nm. **e** Measured co-polarized transmission spectra of the fabricated device. **f**–**h** Measured intensity profiles of the device at the focal plane. The operating wavelengths are **f** 660 nm, **g** 532 nm, and **h** 473 nm. **i** Modulation transfer function (MTF) of the see-through metalens for several wavelengths. The blue dashed line represents the diffraction limited MTF of the see-through metalens for the wavelength of 473 nm

As discussed in the previous section, a transmission-type eyepiece with a high NA and a large area is required to successfully achieve a wide FOV. In this research, a dielectric metasurface with arbitrarily engineered anisotropy throughout the entire visible region is elaborately designed at the subwavelength scale to satisfy the conditions described above for the transmission-type eyepiece. A schematic illustration of the unit cell of the proposed metasurface is presented in Fig. 2a. Theoretically, the rectangular dielectric nanorod with an arbitrary orientation angle can be modeled as a Jones Matrix *T*. Consequently, the complex transmittance of the nanorod for incident circularly polarized light can be expressed as follows (see details in Supplementary Note 2):

1 $$T\left| \sigma \right\rangle = \frac{{t_{\mathrm{l}} + t_{\mathrm{s}}}}{2}\left| \sigma \right\rangle \, + \,\frac{{t_{\mathrm{l}} - t_s}}{2}e^{ - j2\sigma \theta }\left| { - \sigma } \right\rangle,$$

where the parameter *σ* is selected to be +1 or −1 for right or left circular polarization, and *t*_l_ and *t*_s_ are the complex transmission coefficients for linearly polarized light along the longer and shorter axes of the nanorod, respectively. As shown in the equation, the transmitted light consists of two components which are a co-polarized component (*σ*) and a cross-polarized component (−*σ*), and the cross-polarized component experiences a relative phase shift (2*σθ*) that is proportional only to the orientation angle (*θ*), also known as the Pancharatnam-Berry phase or geometric phase.

It is notable that the coefficients (*t*_l_ and *t*_s_) are related to anisotropy of the nanorod, and they can be controlled by varying the dimensions of the nanorods, i.e., the longer and shorter axis lengths of rectangular nanorods of a suitable thickness. For example, if the nanorod is an isotropic optical element that means *t*_l_ and *t*_s_ are identical, the cross-polarized component will be vanished. On the other hand, if the coefficients have reversed phase components with identical amplitudes, the co-polarized component will be vanished. Hence, Eq. (1) indicates that we can design the co-polarized and cross-polarized transmittance amplitudes independently when *t*_l_ and *t*_s_ can be arbitrarily specified, which plays an important role in designing the see-through metalens for AR imaging. Notably, the geometric phase is a purely geometrical effect, leading to broadband characteristics with a phase shift tendency that is independent of the wavelength of the light. To design a metasurface that acts as a spherical lens with a focal length of *f* for the cross-polarized transmission mode, each unit cell of the metasurface is designed by encoding a spatial phase distribution with the following relation:

2 $$\theta \left( {x,y} \right) = \frac{\pi }{\lambda }\left( {f - \sqrt {x^2 + y^2 + f^2} } \right),$$

where *x* and *y* are the positions along the *x* and *y* axes, respectively, and *θ*(*x, y*) is the orientation angle of the nanorod at the position (*x*, *y*). The phase of the co-polarized transmitted light is independent of the orientation angle, so the spatial phase distribution of the co-polarized transmitted light will be constant over the entire region. Thus, the device acts as transparent glass for the co-polarized transmission mode even as it acts as a lens for the cross-polarized transmission mode. Previously, most researchers have attempted to remove this co-polarized component because the co-polarized component has been regarded as critical noise interfering with the cross-polarized component. By contrast, in our work, the co-polarized component plays an important role in the AR display. Consequently, the proposed metalens possesses both see-through and lensing properties according to the polarization.

To make this device suitable for use in an AR display system, we further expand the concept to a see-through metalens for multiple types of incident circularly polarized light with different handednesses. Figure 2b illustrates the optical behavior of the see-through metalens encoded with the spatial phase distribution given in Eq. (2) when circularly polarized light of a given handedness (*σ*) or its opposite counterpart (−*σ*) is incident on the device. For incident light with a handedness of *σ*, the device acts as a convex lens for cross-polarized transmission and as transparent glass for co-polarized transmission. Conversely, for the opposite handedness (−*σ*), the device acts as a concave lens for cross-polarized transmission (*σ*) but still as transparent glass for co-polarized transmission (−*σ*). This behavior is due to the sign reversal of the encoded spatial phase distribution induced by the reversed handedness of the incident circular polarization. Overall, when we consider light with circular polarization of both handednesses normally incident on the see-through metalens, the transmitted light will consist of four components, including a converged light component (with *σ*), a diverged light component (with *−σ*), and two directly transmitted light components of both handednesses. If we block all components with a handedness of −*σ* by using a circular polarizer, then two components of converged and directly transmitted light will remain: the incident light with a handedness of *σ* is converged, and its counterpart with the opposite handedness of –*σ* passes through the device. By applying this strategy, AR imaging can be achieved in which the virtual information is circularly polarized with a handedness of *σ* and the light from the real-world scene has the opposite handedness of −*σ*. Thus, a transmission-type eyepiece can be realized by using this sophisticated concept.

To verify the proposed meta-atom design, a commercial tool (COMSOL) based on the finite element method (FEM) was used (see the Methods section for details). To realize the proposed meta-atom design, poly-crystalline silicon (Poly-Si) was employed due to its CMOS compatibility, which makes the proposed structure feasible for mass production. The nanorod dimensions were designed by considering the resulting broadband characteristics. Specifically, the nanorod length and width are related to the spectra for both the co-polarized and cross-polarized transmission coefficients. Notably, the co-polarized transmission coefficient is more important than the cross-polarized one when the see-through metalens is to be used in an AR display. Since the power of the virtual information can be adjusted, whereas it is impossible to regulate or enhance the power of the light from the real-world scene, it is essential to design the co-polarized transmission to be as high and uniform as possible throughout the spectrum. Therefore, we carefully designed the length and width of the nanorods with a focus on controlling and improving the co-polarized transmission throughout the spectrum. As a result, the nanorods were designed to have a length of 220 nm, a width of 60 nm, and a thickness of 100 nm, and the period of the unit cells was set to 400 nm (see the Supplementary Note 4 and Supplementary Fig. 4 for details). Figure 2c shows the simulated phases of both the co-polarized and cross-polarized transmitted light as functions of the orientation angle for wavelengths corresponding to three colors: red (660 nm), green (532 nm) and blue (473 nm). The phase of the cross-polarized transmitted light is linearly related to the orientation angle, whereas the phase of the co-polarized transmitted light is constant with respect to the orientation angle.

A prototype of our see-through metalens was fabricated by using nanoimprint technology, and the fabricated sample is shown in Fig. 2d (see the Methods section for details). The metalens was fabricated with a diameter of 20 mm to provide a wide FOV. In the right side of Fig. 2d, a scanning electron microscope (SEM) image of the fabricated sample is presented. It is significantly notable that we chose nano-imprinting technology to demonstrate the feasibility of the proposed device for mass production; by contrast, almost previous works on metasurface applications have employed only electron-beam lithography fabrication, which is quite far from being suitable for mass production due to its considerable expense and long fabrication time. Although the resolution of nano-imprinting is lower than that of electron-beam lithography, the SEM image shows that the nanostructures were sufficiently precisely fabricated, confirming the feasibility of this fabrication method. Figure 2e shows the polarization conversion efficiencies of the proposed nanorods for co-polarized transmission. The efficiencies are approximately 79% for co-polarized transmission at wavelengths in the red, green and blue color regions. Moreover, the co-polarized spectrum shows the desired broadband characteristic of the proposed meta-atom, with a regular and broad slope throughout the entire visible region. As previously explained, we focused our design on the co-polarized transmittance, which is directly related to the power of the light from the real-world scene; consequently, the represented spectrum ensures a clear view of the real-world scene. The calculated efficiencies for the cross-polarized components are 29, 6, and 5% for red, green, and blue wavelengths, and the measured efficiencies are 12, 9, and 2.5% for the same wavelengths, respectively. The differences between calculation and measurement can be improved using more accurate fabrication process. The absorption for those wavelengths is 1, 8.2, and 21.4%, respectively, and it can be dramatically reduced using other low-loss dielectric including titanium dioxide or silicon nitride while we used silicon to consider CMOS-compatible mass production and industrialization. Further developments on metasurfaces using low-loss dielectric materials like titanium dioxide, gallium or silicon nitrides could enhance the efficiency and reduce the absorption. A compromise of the co-polarized efficiency would also make the cross-polarized efficiency higher for providing a virtual image clearly under the strong sunlight.

As explained, all of the geometric phase shifts are constant for different wavelengths. Consequently, light of all different wavelengths experiences the same phase delay, which results in variation of the focal length. The higher NA is required to provide the wider FOV in a see-through near-eye display, so the NA of the metalens was designed to be maximum under this condition. The maximum NA is related to Nyquist’s sampling theory; the period of the unit cells determines the sampling frequency, with a higher sampling frequency resulting in a higher NA. In our case, the structure has a period of 400 nm, and this value corresponds to a maximum NA of 0.8. In our device, the focal lengths for red, green and blue light were set to 12.9, 16, and 18 mm for wavelengths of 660, 532, and 473 nm, respectively. In other words, the NAs of the see-through metalens are 0.61, 0.53, and 0.49 at these wavelengths. Figure 2f, g, h depict experimental demonstrations of the fabricated metalens using lasers of three different colors at the wavelengths, respectively. Based on the microscopy set-up, the intensity at the focal plane under the normal incidence was captured for each wavelength, and the lower row of Fig. 2f–h shows the intensity profiles near the focal point in one-dimensional space (see Supplementary Note 7 and Supplementary Fig. 7 for the experimental set-up). These findings confirm the good focusing performance of the see-through metalens. Although our metalens system is based on the normal incidence, it is also required to consider the slanted incident angles, which affects the image quality of large FOV and eyebox (See Supplementary Note 8 and Supplementary Figs. 8 and 9 for focal points for oblique incidence, and Supplementary Note 9 and Supplementary Fig. 10 for image uniformity of the virtual image). Monochromatic aberration for the slanted incident angles occurs, which hinders image quality near large FOV. It has been shown that the doublet metalens could correct this monochromatic aberrations, which can make our see-through metalens reconstruct sharper images around the edges^14,41^. To analyse the imaging quality of the see-through metalens, modulation transfer functions (MTF) for each wavelength were calculated using the point spread functions (PSF) in Fig. 2f–h and are shown in Fig. 2i. The MTF curves show that the see-through metalens can provide sufficient image quality where the calculated MTF from PSF is close to the diffraction limited MTF. Because the cut-off frequency of the MTF is proportional to NA/*λ*, the imaging resolution decreases as the wavelength increases, and this can be more improved by designing a see-through metalens with a higher NA. Another point deteriorating the MTFs is the chromatic aberration of the metalens due to the phase matching condition (see Supplementary Note 11 and Supplementary Fig. 13), which would be corrected using aberration-corrected metalenses or a holographic method with aberration correction.

### Full-color metasurface near-eye display with chromatic correction

**Fig. 3:**
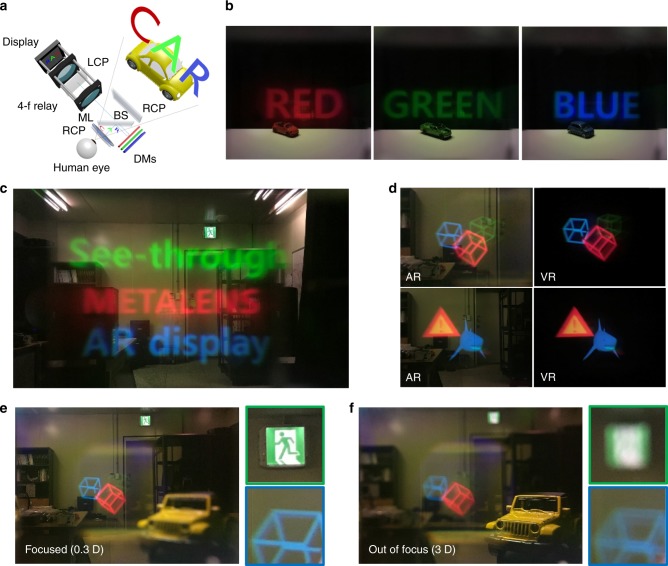
See-through near-eye display with the proposed metalens. **a** Illustration of the prototype. LCP or RCP denotes a left or right circular polarizer, respectively, ML is the metalens, the DMs are dichroic mirrors, and BS is a beam splitter. **b** A representative full-color AR imaging result. All the augmented images are focused simultaneously. **c** Single-color AR images with real objects for red, green, and blue colors. **d** Full-color augmented and virtual images for different contents. AR is the augmented image, and VR is the virtual image. Corresponding movie clip and original images are in Supplementary Movie 1 and Supplementary Figs. 5 and 6. **e**, **f** Demonstration of the floating depth of the image. The floated images are **e** focused at 0.3 diopters and **f** blurred at 3 diopters. Side images in green and blue boxes are part of the original, showing that the emergency light and blue cube are at the same depth. The images shown here were captured by a cell phone

Based on the proposed see-through metalens concept, we designed a prototype of a see-through near-eye display. Figure 3a shows the system configuration for implementing a full-color near-eye display using the proposed metasurface. The system is composed of a beam projector, a 4-f relay system, a beam splitter, dichroic mirrors, circular polarizers and the metalens (see the Methods section for details). It is notable that the see-through metalens is located right in front of the human eye, and the image is positioned within the focal length of the metalens, leading to large FOV of virtual images. In the prototype, we also correct the chromatic aberration of the metalens by varying the imaging position with the wavelength using three dichroic mirrors, which allows us to implement a full-color see-through near-eye display system with a single display device using our metalens (see the Methods section for detials).

Figure 3b shows the experimental results for each color at three wavelengths (660, 532, and 473 nm). Figure 3c shows the full-color representation achieved using the dichroic mirrors in which all the images with different colors are floated in the same depth. Since the spherical aberration is lower than that of a conventional lens due to the nature of the metalens, the image is not much distorted in the edge region despite the wide FOV. Figure 3d shows the augmented images (for AR) and the corresponding virtual images (for virtual reality) for the contents of three cubes or a shark with hazard symbol (see Supplementary Note 5 and Supplementary Fig. 5 for the original images). Since the efficiency of the metalens varies with the wavelength, this variation is compensated on the display side. A movie clip for continuous change of the corresponding images in Fig. 3d is shown in Supplementary Movie 1 with explanation in Supplementary Note 6 and Supplementary Fig. 6. Finally, Fig. 3e, f show the location to which the image is floated, as demonstrated by changing the focus of the camera. As seen in Fig. 3e, the virtual information (three cubes) is clearly displayed when the camera is focused at 3 m depth (0.3 diopters), but it is blurred when the camera is focused at 0.3 m depth (3 diopters), as shown in Fig. 3f. The side figures within the green or blue boxes are part of the original, showing that the cube and the emergency light on the laboratory wall are in the same depth position. The overall efficiency of the prototype system was measured as 1%. We believe that this can be improved by development of more efficient metalenses as we discussed before.

**Fig. 4:**
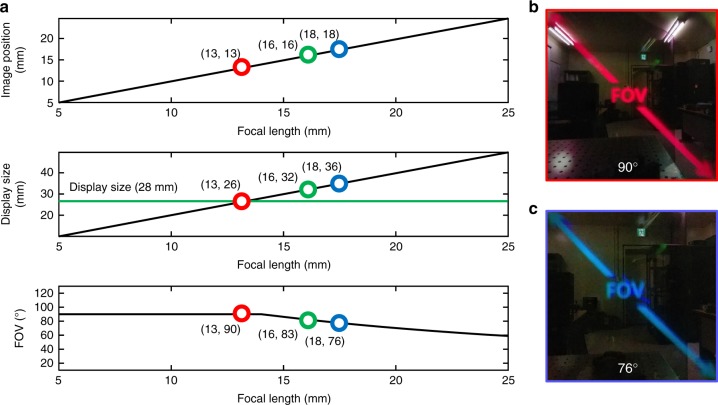
Theoretical and experimental demonstration of the FOV and form factor. **a** Relationship between the focal length and system performance (form factor and FOV). Achievable FOV as a function of the focal length at red (660 nm, 13 mm), green (532 nm, 16 mm) and blue (473 nm, 18 mm) wavelengths. The red, green, and blue points represent system performance of the prototype according to each wavelength. **b**, **c** Achievable FOVs corresponding to the red and blue points in **a**. The FOV is 90° in **b** and 76° in **c**. The wide-FOV images shown here were captured by a cell phone (V20, LG)

Figure 4a shows the FOV and form factor of the proposed system. The greatest advantage of using the proposed metalens in a see-through near-eye display is that the eyepiece can be placed just in front of the eye, so the FOV is solely determined by the eye relief distance and the lens aperture. Figure 4a shows the simulated system performances of prototype. The lens aperture is 20 mm and the focal lengths are 12.9, 16, and 18 mm for the three wavelengths. The fixed eyebox and the eye relief of 10 mm is set to synchronize with the actual prototype specification. Our prototype can have maximum FOV of 90° because the lens aperture is limited to 20 mm. The minimum display size to provide the maximum FOV depends on the focal length. In other words, the required display size differs according to the wavelength in metalens. For red color (660 nm), the 26 mm image size is required to provide the maximum FOV, while the 36 mm display size is required for a blue color (473 nm). Therefore, the display size of 36 mm is required to provide a full color image with the maximum FOV of 90°. However, in our prototype, we use a beam splitter and display with a size of 28 mm; Thus, we can provide an FOV of 90° for red light and an FOV of 76° for blue light, as shown in Fig. 4a, and corresponding AR images are shown in Fig. 4b, c, respectively. This is similar to the display limit mentioned in Fig. 1e. For full-color imaging, all three colors (red, green, and blue) should be represented simultaneously, and the common region in which all three colors can be displayed is the same as the blue display region, which has the narrowest FOV. Therefore, our prototype achieves an FOV of 90° for monochrome imaging and an FOV of 76° for full-color imaging. The corresponding FOVs are verified in Fig. 4b, c. The wide FOV of this system relative to its form factor is possible due to the novel optical properties of the metalens, especially the polarization selectivity and the high NA with a large lens aperture. Consequently, our proposed system can provide a wider FOV than any other conventional near-eye display systems for AR.

Although our system successfully provides a wide FOV in a suitable compactness compared to other systems, the compactness of our system remains to be improved. For example, several systems using HOE have shown more compact systems, but with a very small eyebox due to the angular selectivity of the volume grating^33^. In our case based on the half mirror, there are several points that interfere with compactness but can be improved in the future. First, the chromatic dispersion of our metalens makes the system bigger because it requires the use of three dichroic mirrors to implement the full-color imaging. For this, the further development of an achromatic metalens for AR applications could be a possible solution. As mentioned before, the current achromatic metalenses are not capable of providing clear see-through property as well as large aperture with high NA due to limited control of dispersion. However, these approaches are enough to show the great potential of the achromatic metalens in AR application^16,17^. With the development of this achromatic metalens for the see-through metalens, the volume of the proposed system can be mitigated. Next, a holographic method can also be a solution for this. A method of sending virtual images with different focal lengths at the display stage can be considered in the further study, and the holographic technology can make it possible to reconstruct different wavefronts to compensate the different focal lengths of the metalens. This method does not need the dichroic mirrors either, so we believe that this approach is one of the proper solutions for the chromatic aberration of the current see-through metalens. Finally, a half mirror in our system can be improved to make the system smaller. Recent advances in metasurfaces have shown that the various works for developments of multifunctional mirrors are going on. For example, the reflection on the metasurface can be designed differently from the general reflection and the high reflection angle from the metasurface can be achieved^42^. This high reflection angle with the designed reflection relationship will make the AR system more compact owing to the reduction of the tilted angle of the half mirror.

## Discussion

In summary, we have proposed a metasurface application to realize a see-through near-eye display system with a wide FOV. Utilizing a sophisticatedly engineered anisotropic nanostructure, we implemented a see-through metalens that functions as transparent glass for light from real-world scenes and as an eyepiece for floating virtual information. This transmission-type eyepiece can achieve a wider FOV than is possible in previously developed systems based on conventional optics. To satisfy the criteria for AR display, we also optimized the see-through metalens to have a uniform transmittance spectrum for co-polarized transmission, resulting in a clear view of the real-world scene without chromatic distortion. Via nano-imprinting technology, a prototype metalens with a lens aperture of 20 mm and a high NA of 0.61 was fabricated to demonstrate the ability to achieve a wide FOV. Consequently, a see-through augmented image with a wide FOV of 90° was experimentally achieved in the proposed system. The chromatic aberration, which has been a persistent disadvantage of metalenses, was also corrected with multi-layered dichroic mirrors to successfully achieve a full-color AR image. By virtue of the compactness and wide FOV of the proposed system, highly immersive AR glasses as a socially acceptable platform are no longer an unattainable dream. Moreover, our research represents an attempt not just to improve the performance of existing AR displays but also to advance to the next generation of optical devices through the introduction of novel optics technology (meta-optics). We expect this innovative and initiative approach will pave the way towards a new turning point across the realms of consumer products, industry and scientific research and will have numerous applications in the fields such as future optical displays, computer vision, wearable electronics, biological imaging, medical devices, and optical microscopy.

## Methods

### Numerical simulation

The results presented in Fig. 3 were calculated via the finite element method (FEM) using commercial software (COMSOL Multiphysics 5.2). All simulations were performed within an *xyz* space with dimensions of 400 nm × 400 nm × 3 μm. Periodic boundary conditions were applied along the *x* and *y* axes, with a period of 400 nm on both axes. The refractive indices of the materials comprising the meta-atoms were specified based on the values from reference^43^. To calculate the phase information for both co-polarized and cross-polarized transmission, the corresponding transmission coefficients were computed for all orientation angles.

### Device fabrication

The metasurface was fabricated by nanotransfer lithography process. It is noted that schematic of the overall process is depicted in Supplementary Note 12 and Supplementary Fig. 14. Firstly, the polymer stamp was prepared using polyurethane-acrylate (PUA) MINS-311 RM (Minuta Technology Co.) imprint resin and the silicon master fabricated by electron-beam lithography. The Au, Cr and SiO_2_ were deposited on the prepared polymer stamp using electron-beam evaporator (Daeki Hi-Tech Co.) at 1 Å/s and high vacuum condition. Secondly, the poly-crystalline silicon (Poly-Si) was deposited on the quartz wafer substrate using low pressure CVD system. Then, the N-[3-(trimethoxysilyl)propyl]ethylenediamine with solvents (propane-1,3-diol and di(propylene glycol) methyl ether) as an adhesive layer was coated on the Poly-Si deposited quartz wafer substrate using spin coating system. The polymer stamp with Au, Cr, and SiO_2_ was placed over the prepared quartz substrate with adhesive and Poly-Si, then pressure was applied to transfer the deposited materials from the polymer stamp using roll-to-plate system (Eastern Engineering). Owing to formed covalent bonding between SiO_2_ and adhesive^44^, Au, Cr, and SiO_2_ were transferred to the substrate, and the transferred Cr was used as a hard mask to etch Poly-Si layer. After etching process, the Cr was removed using Chromium etchant from Sigma-Aldrich. Finally, thin residues above the silicon metasurface are removed by etching process and the metasurface device was fabricated.

### Prototype configuration of the metasurface near-eye display

For experimental demonstration, we designed a prototype of full-color near-eye display using the proposed metasurface as represented in Fig. 3a. A beam projector based on the display panel with the halogen lamp was used in our prototype with natural density filter to resolve safety issue for the human eye. As shown in Fig. 1b, the see-through metalens is located right in front of the human eye, and the image is positioned within the focal length of the metalens. We use a beam splitter and a 4-f relay system to clearly demonstrate the feasibility of the proposed concept. A waveguide and a transparent screen including a diffuser holographic optical element (DHOE) are also good candidates for conveying the image to its proper position^40,45^. For the utilization of the polarization-selective property of the metalens, three polarizers exist in the system. First, a left circular polarizer is placed in front of the display. It polarizes the virtual information from the display into the left circularly polarized state. The virtual information with left circular polarization is modulated by the metalens into left and right circularly polarized components. As shown in Fig. 2b, the right circularly polarized component of the virtual information is floated to the desired depth by the metalens, while the left circularly polarized component passes through the metalens. The transmitted left circularly polarized component is blocked by a right circular polarizer in front of the metalens, so that only the floated virtual information with right circular polarization is observed. Meanwhile, the light from the real-world scene is right circularly polarized by a right circular polarizer behind the beam splitter and enters the metalens. As shown in Fig. 2c, this right circularly polarized light is modulated by the metalens into a left circularly polarized component with phase modulation and a right circularly polarized component is transmitted without modulation. The right circular polarizer blocks the unnecessary left circularly polarized component of the light from the real-world scene. Ultimately, this polarization control allows the user to observe a clear image of the real-world scene that is not modulated by the metalens.

Notably, the see-through metalens has different focal lengths at different wavelengths, so it cannot realize full-color imaging with a single-layer image, as shown in Fig. 1c. Therefore, we correct the chromatic aberration of the metalens by varying the imaging position with the wavelength using three dichroic mirrors (for the spectra of the dichroic mirrors, the source spectrum, and analysis of the effect of bandwidth on MTF, see Supplementary Note 10, Supplementary Figs. 11 and 12). This allows us to implement a full-color see-through near-eye display system with a single display device using our metalens. Although several achromatic metalenses were proposed and show their possibilities, they are still not suitable for use in AR applications due to the limited sizes and NAs as well as limited anisotropy^16,17^. In our prototype, since the desired float depth is 3 m (0.3 diopters) and the focal lengths of the metalens are 12.9, 16, and 18 mm at the target wavelengths (660, 532, and 473 nm, respectively), the corresponding images should be located 12.9, 16 and 18 mm in front of the metalens, respectively (for the detailed specifications of the system configuration, see the Supplementary Note 3 and Supplementary Fig. 3).

## Electronic supplementary material

Supplementary Information

Supplementary Movie 1

Description of Additional Supplementary Files

## Data Availability

The data that support the findings of this study are available from the corresponding author upon reasonable request.
