# Supplementary material for: Metasurface eyepiece for augmented reality
Source: Nat Commun. 2018 Nov 1;9:4562. doi: 10.1038/s41467-018-07011-5 (PMC6212528; doi:10.1038/s41467-018-07011-5)
Supplement: Supplementary file 1 — Supplementary Information [file 41467_2018_7011_MOESM1_ESM.pdf]

Supplementary Information

Metasurface eyepiece for augmented reality

*Gun-Yeal Lee[1, †], Jong-Young Hong[1, †], SoonHyoung Hwang[2], Seokil Moon[1], Hyeokjung Kang[2], Sohee Jeon[2], Hwi Kim[3], Jun-Ho Jeong[2], and Byoungho Lee[1, *]*

[1] School of Electrical and Computer Engineering, Seoul National University, Gwanakro 1, Gwanak-Gu, Seoul 08826, South Korea

[2] Nano-Convergence Mechanical Systems Research Division, Korea Institute of Machinery and Materials, Gajeongbuk-ro 156, Youseong-gu, Daejeon 34103, South Korea

[3] Department of Electronics and Information Engineering, Korea University, 2511 Sejong-ro, Sejong 30019, South Korea

*Corresponding author email: byoungho@snu.ac.kr

Supplementary Note 1. Parameter characterizations and analysis

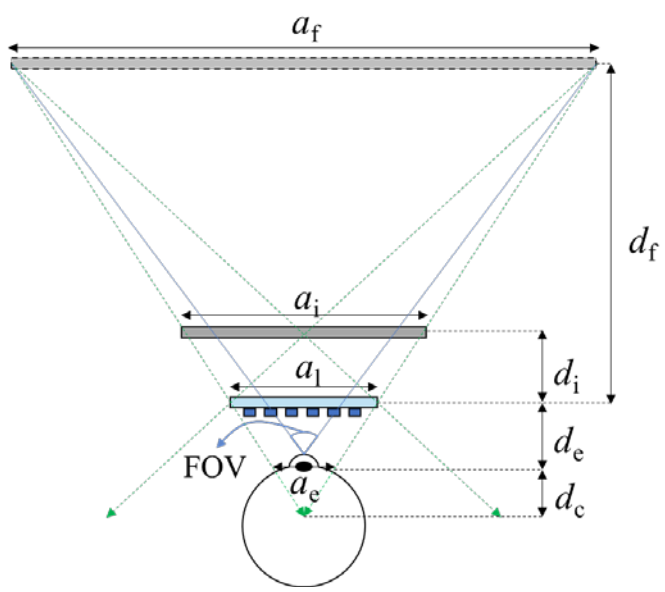

Supplementary Figure 1. Schematic for specific parameters in AR near-eye display with a transmission-type eyepiece.

The eye relief (d_e), desired floating depth (d_f), lens aperture (a_l) and eyebox (a_e) are fixed variables in AR glasses design. The FOV and required display size (a_i) are presented as follows:

$$d_c = \frac{a_e d_e}{(a_l - a_e)}, \tag{1}$$

$$a_f = \frac{a_e(d_c + d_e + d_f)}{d_c}, \tag{2}$$

$$d_i = \frac{f d_f}{d_f + f}, \tag{3}$$

then, $a_i = \frac{a_f d_i}{d_f}$, and $\mathrm{FOV} = 2\tan^{-1}\left(\frac{a_f}{d_e + d_f}\right)$, where the a_i is required display size.

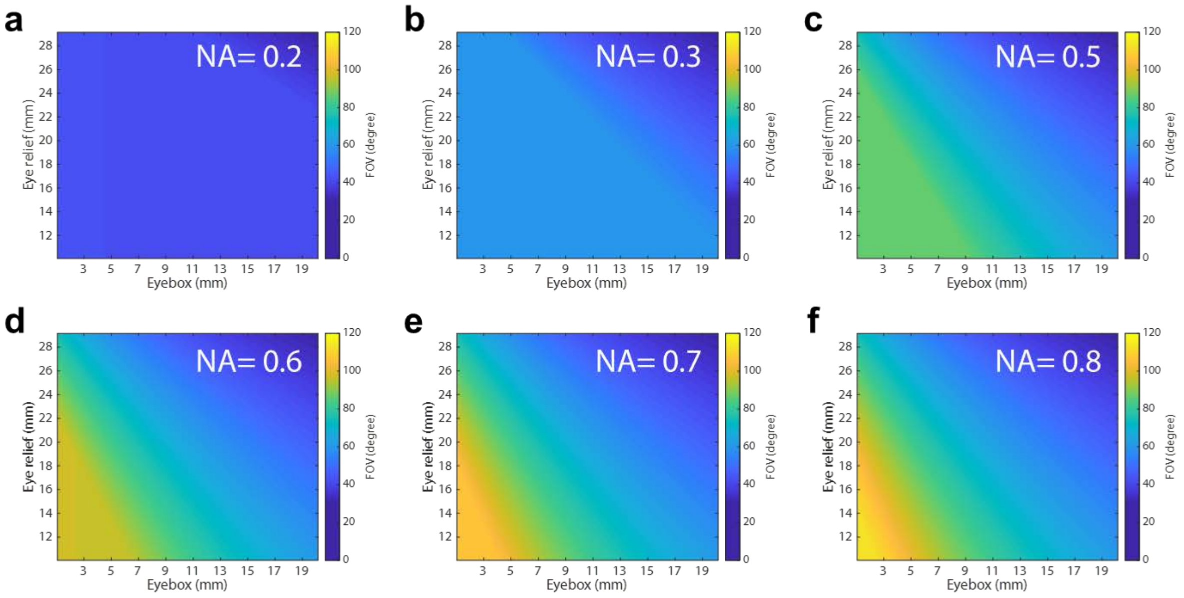

Supplementary Figure 2. System performance of the transmission type see-through near-eye display. (a-f) The numerical calculations show the FOV according to eyebox and eye relief where the lens aperture is set to 35 mm. Several numerical apertures (NAs) including (a) 0.2, (b) 0.3, (c) 0.5, (d) 0.6, (e) 0.7 and (f) 0.8 are considered. As shown in the figures, the wide eyebox and long eye relief are in trade-off relationship with the FOV. Moreover, the higher the NA or the shorter the focal length within the same lens aperture provides the higher viewing angle under the same conditions (i.e. same eyebox and eye relief).

Supplementary Note 2. Jones matrices for an arbitrarily anisotropic nanorod

In this section, we show that the complex transmittance of the nanorod can be described as Equation (1) in the main text. An arbitrarily anisotropic nanorod can be represented by a Jones matrix within the coordinates consisting of a longer optical axis and a shorter optical axis, and the Jones matrix can be described as follow:

$$J = \begin{pmatrix} t_l & 0 \\ 0 & t_s \end{pmatrix}, \tag{4}$$

where the t_l and t_s are the complex coefficients for longer and shorter optical axis, respectively. Therefore, using the coordinate rotation, the Jones matrix of anisotropic nanorods having arbitrary orientation is

$$T = R(-\theta)J\,R(\theta) = \begin{pmatrix} \cos\theta & \sin\theta \\ -\sin\theta & \cos\theta \end{pmatrix}\begin{pmatrix} t_l & 0 \\ 0 & t_s \end{pmatrix}\begin{pmatrix} \cos\theta & -\sin\theta \\ \sin\theta & \cos\theta \end{pmatrix}, \tag{5}$$

where θ is the orientation angle of the nanorod, and R(θ) is the rotation matrix. In case of the circularly polarized incidence with σ (where σ=1 or -1 for right or left circular polarization, respectively), the complex transmittance from the nanorod can be calculated using the Jones matrix T as follows:

$$E_t = T\left|\sigma\right\rangle = \begin{pmatrix} t_l\cos^2\theta + t_s\sin^2\theta + j\sigma(t_s - t_l)\sin\theta\cos\theta \\ (t_s - t_l)\sin\theta\cos\theta + j\sigma(t_l\sin^2\theta + t_s\cos^2\theta) \end{pmatrix} = \frac{t_l + t_s}{2}\left|\sigma\right\rangle + \frac{t_l - t_s}{2}e^{\mp j2\sigma\theta}\left|-\sigma\right\rangle, \tag{6}$$

where the Jones vectors for circular polarization is represented as $\left|\pm\sigma\right\rangle = \begin{bmatrix}1 & \pm j\sigma\end{bmatrix}^T/\sqrt{2}$. As shown in the right side of Equation (S3), we can see the complex transmittance for circularly polarized incidence is composed of two orthogonal components with their own complex amplitudes while the phase delay through the orientation angle only exists in cross-polarized components.

Supplementary Note 3. System configurations of the prototype setup

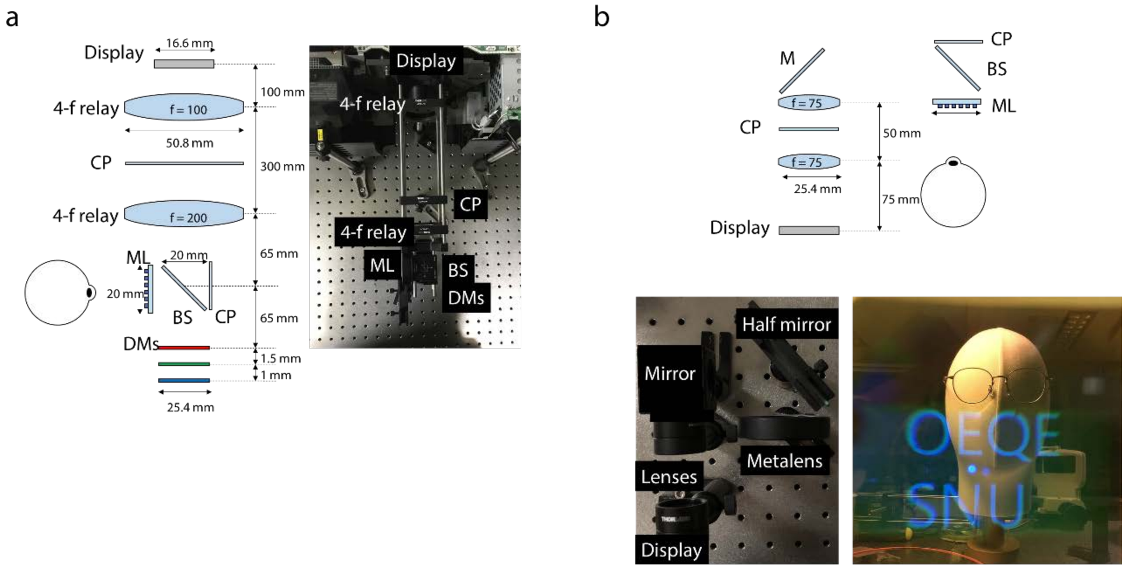

Supplementary Figure 3. The detailed experimental specification and setups (a) a benchtop prototype and (b) a compact head-mounted display version. ML is a see-through metalens, M is a mirror, BS is a beam splitter, DMs are dichroic mirrors with their own transmission spectra, and CP is a circular polarizer.

Figure S3(a) shows the benchtop prototype used in our experiments. We use the spatial light modulator (SLM) in the Sony projector. To show wide FOV of the proposed see-through near eye display, the lenses with focal length of 100 mm and 200 mm are used and magnifies the SLM of projector 2 times. The lens aperture is 20 mm and half mirror of 28 mm by 20 mm is used for beam split. Figure S3b(b) shows the compact version of the proposed see-through near-eye display. The bottom left figure is the working prototype and bottom right figure shows blue monochromatic test results.

Supplementary Note 4. Parametric optimizations of the metasurface

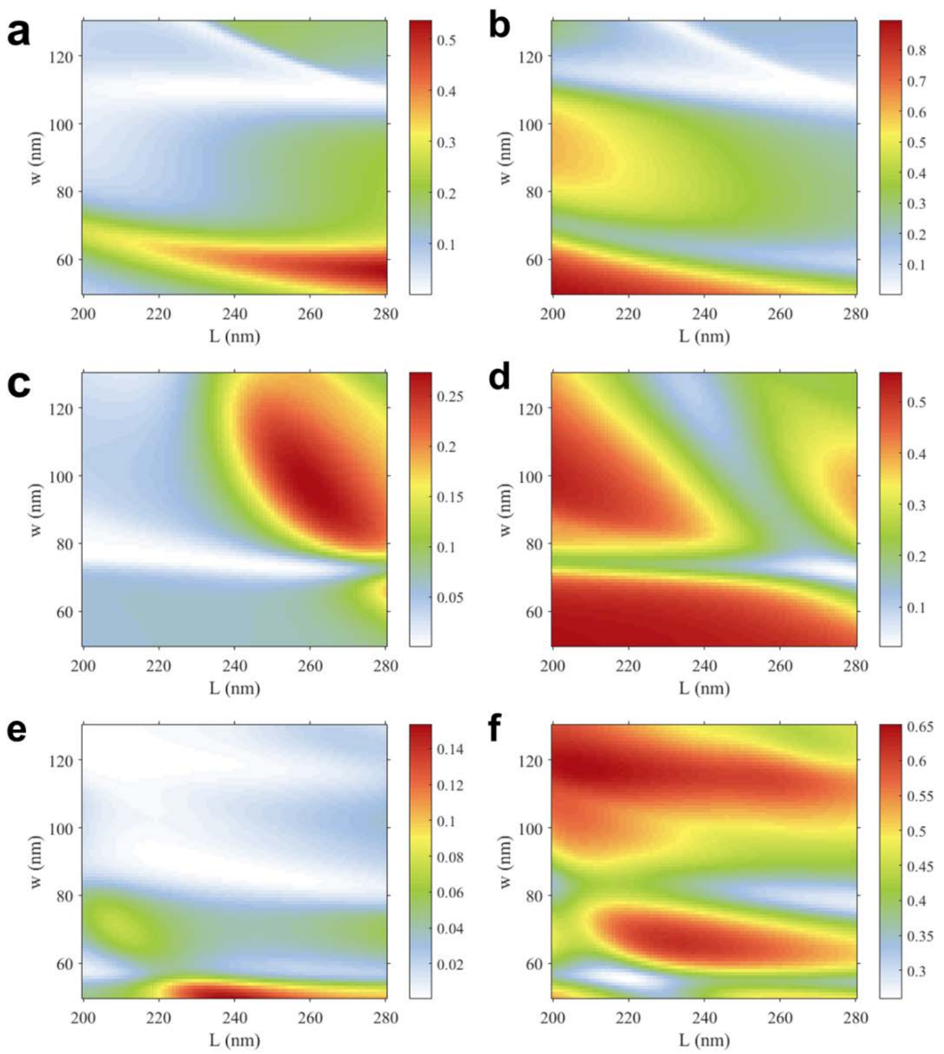

Supplementary Figure 4. Calculated transmission efficiency for co- and cross-polarized light with several wavelengths. Results of two dimensional parametric optimizations while the length (*L*) and width (*w*) of the nanorods are used as variables. As explained in the main manuscript, there are two different components in the transmission part with co- or cross-polarized light. The calculated transmission efficiencies for (a, c, e) cross-polarized and (b, d, f) co-polarized transmission are represented in two dimensional plots with respect to the length and width of the nanorods. Three wavelengths are used in the calculation, which are (a, b) 660 nm, (c, d) 532 nm, and (e, f) 473 nm corresponding to the colours of red, green, and blue, respectively.

Supplementary Note 5. Original images of the objects

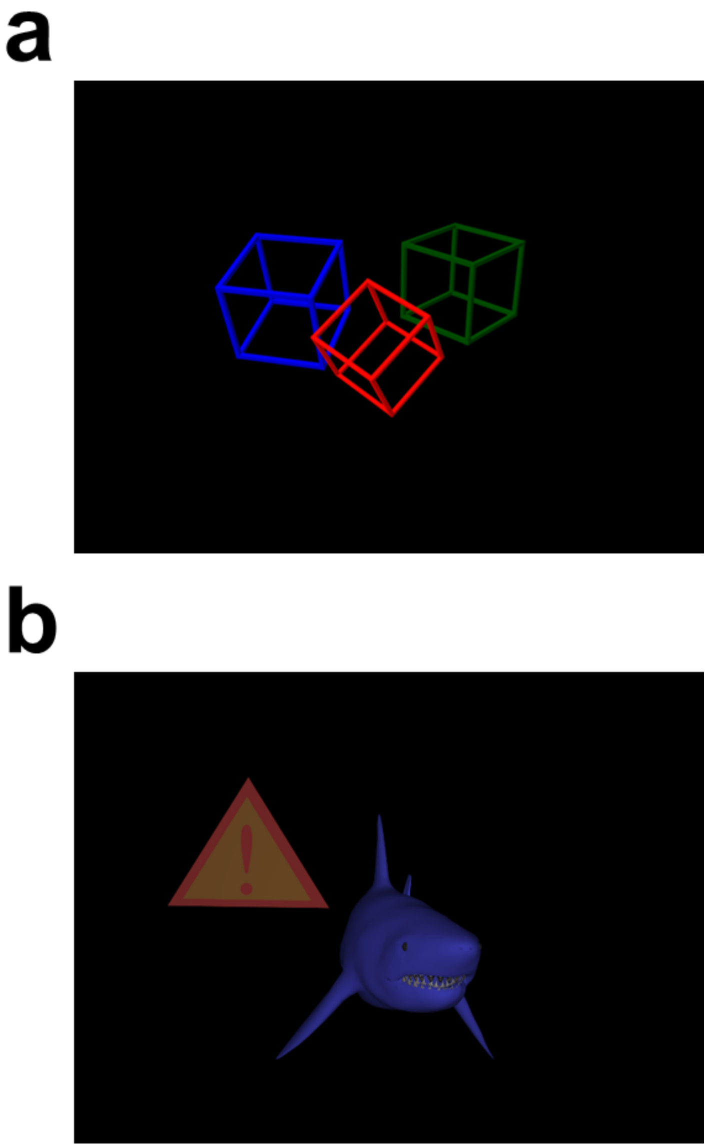

Supplementary Figure 5. Original images used in the experiments represented in Figure 3d. (a) Three cubes and (b) a shark with an emergency mark are corresponding to the experimental results in the upper and lower results in Fig. 3d, respectively. It is notable that the colour densities of the original images are adjusted to get desired colour images while the colour intensities of the see-through metalens are not uniform within the entire visible region. All the contents used in our work are home-made images.

Supplementary Note 6. Descriptions of the video clip

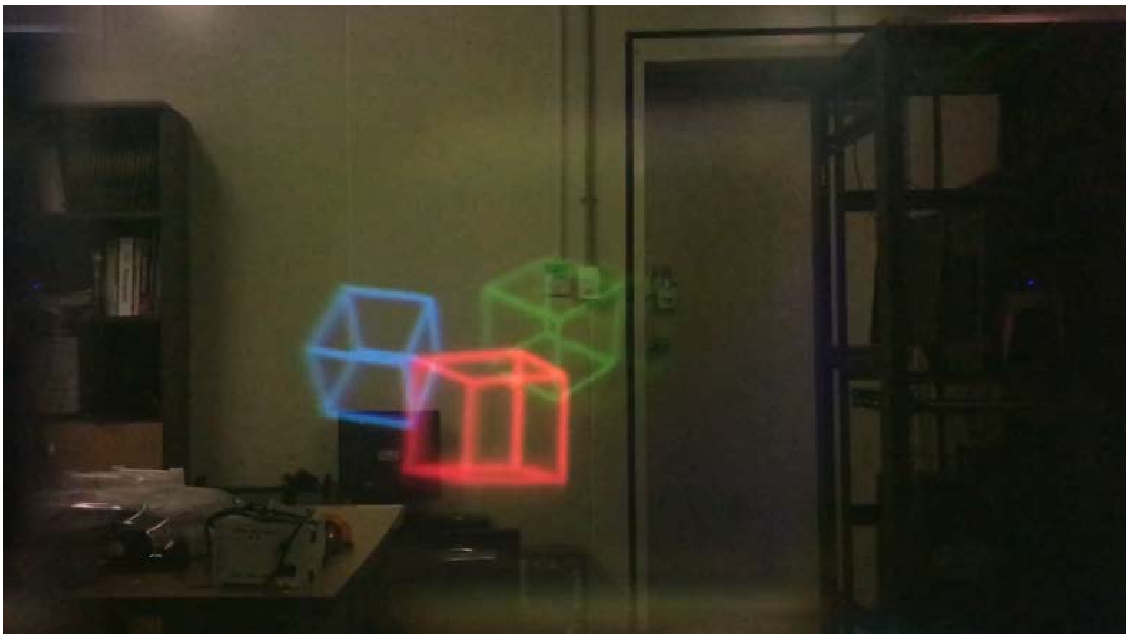

Supplementary Figure 6. Explanation on Supplementary Movie 1. Video clips are experimentally recorded for continuous changes of the augmented images with respect to time. Supplementary Movie 1 is corresponding to Fig. 3d in the main article. In the video, there are two parts. The first part is the video clips for the results in upper part of Fig. 3d for the rotating three cubes. The second part of the video clips is for the results in lower part of Fig. 3d, which is for the moving shark with an emergency mark. The background is the authors' laboratory. These are repeated several times in the entire play time.

Supplementary Note 7. Experimental setup for focal spot measurements

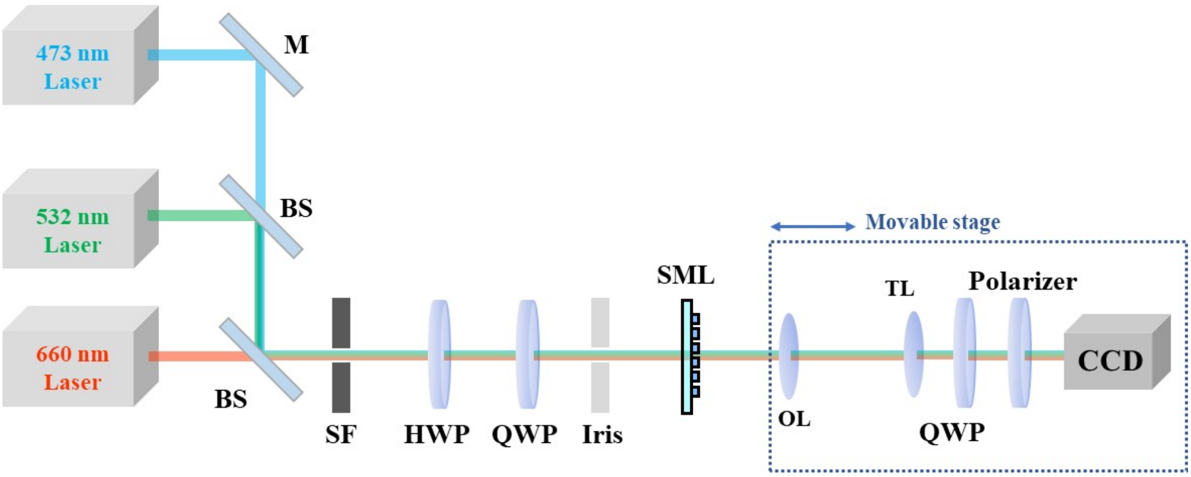

Supplementary Figure 7. Optical setup for experimentally verifying the focusing characteristics of fabricated see-through metalens. Three lasers with the wavelengths of 660 nm (red), 532 nm (green), and 473 nm (blue) are collimated and aligned to a single optical path for convenience. After passing through a spatial filter (SF), a half-wave plate (HWP), a quarter-wave plate (QWP), and an iris, the laser beams with circular polarization enter the see-through metalens (SML) with a diameter of 20 mm. Then the focused beam from the SML is collected by an objective lens (OL; 100x magnification, NA = 0.7) and corresponding tube lens (TL), and comes to the CCD camera after passing through a QWP and a polarizer to only measure the cross-polarized components.

Supplementary Note 8. Focal spots and efficiency for the oblique incidence

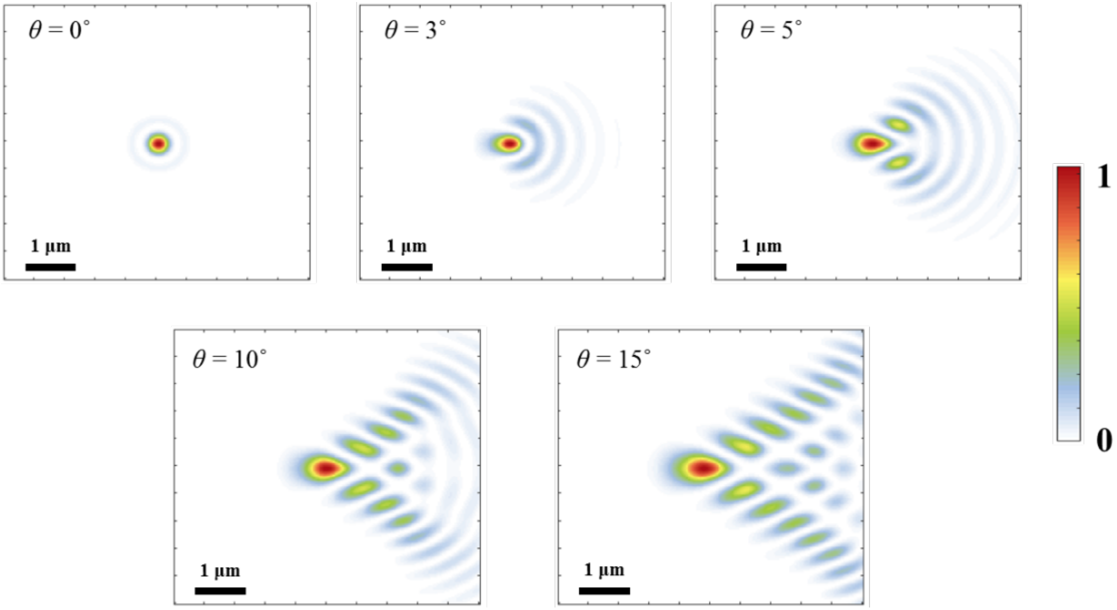

Supplementary Figure 8. Field distributions at the focal points of the see-through metalens for slanted incident angles. The wavelength of the incident beam is 532 nm in all figures. The calculations were performed by using angular spectrum methods based on the phase mask of the proposed metalens.

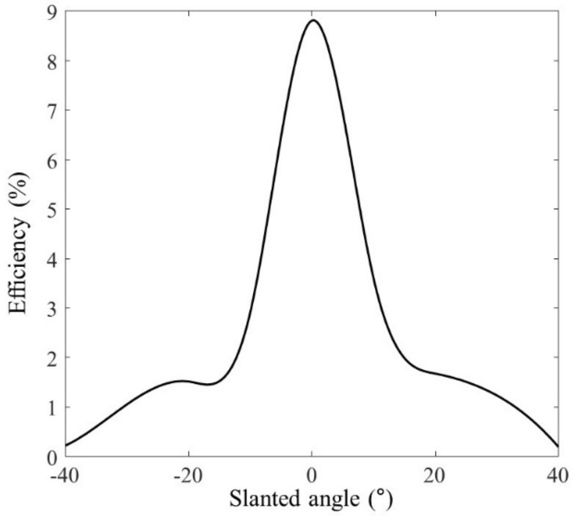

Supplementary Figure 9. Measured focusing efficiency for the slanted incident angles. In the measurement, laser with the wavelength of 532 nm was used. Asymmetry in the graph is due to the measurement errors while the designed metalens is theoretically symmetric.

Supplementary Note 9. Image uniformity of the virtual image

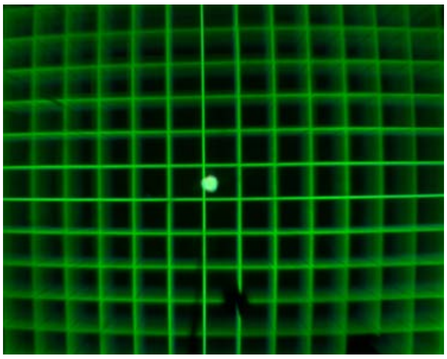

Supplementary Figure 10. Measured virtual images from the see-through metalens. The checker-board image was used to clearly show the uniformity of virtual imaging.

Supplementary Note 10. Spectrums of the source and dichroic mirrors & MTF analysis in terms of the bandwidth

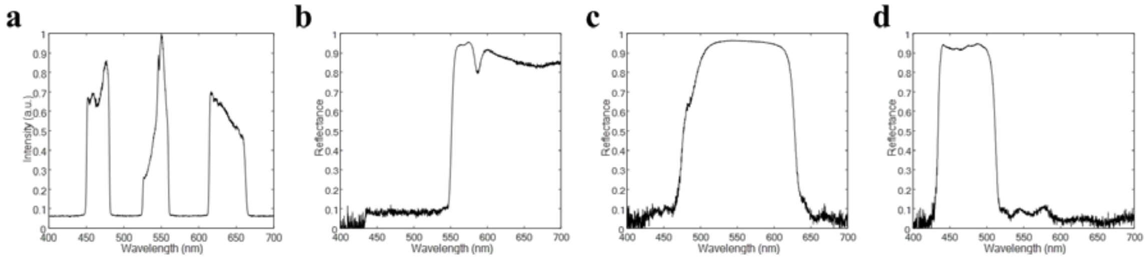

Supplementary Figure 11. Measured spectra for (a) beam projector and (b-d) dichroic mirrors used in the experiments. Three dichroic mirrors for (b) red, (c) green and (d) blue colours were used in our AR system.

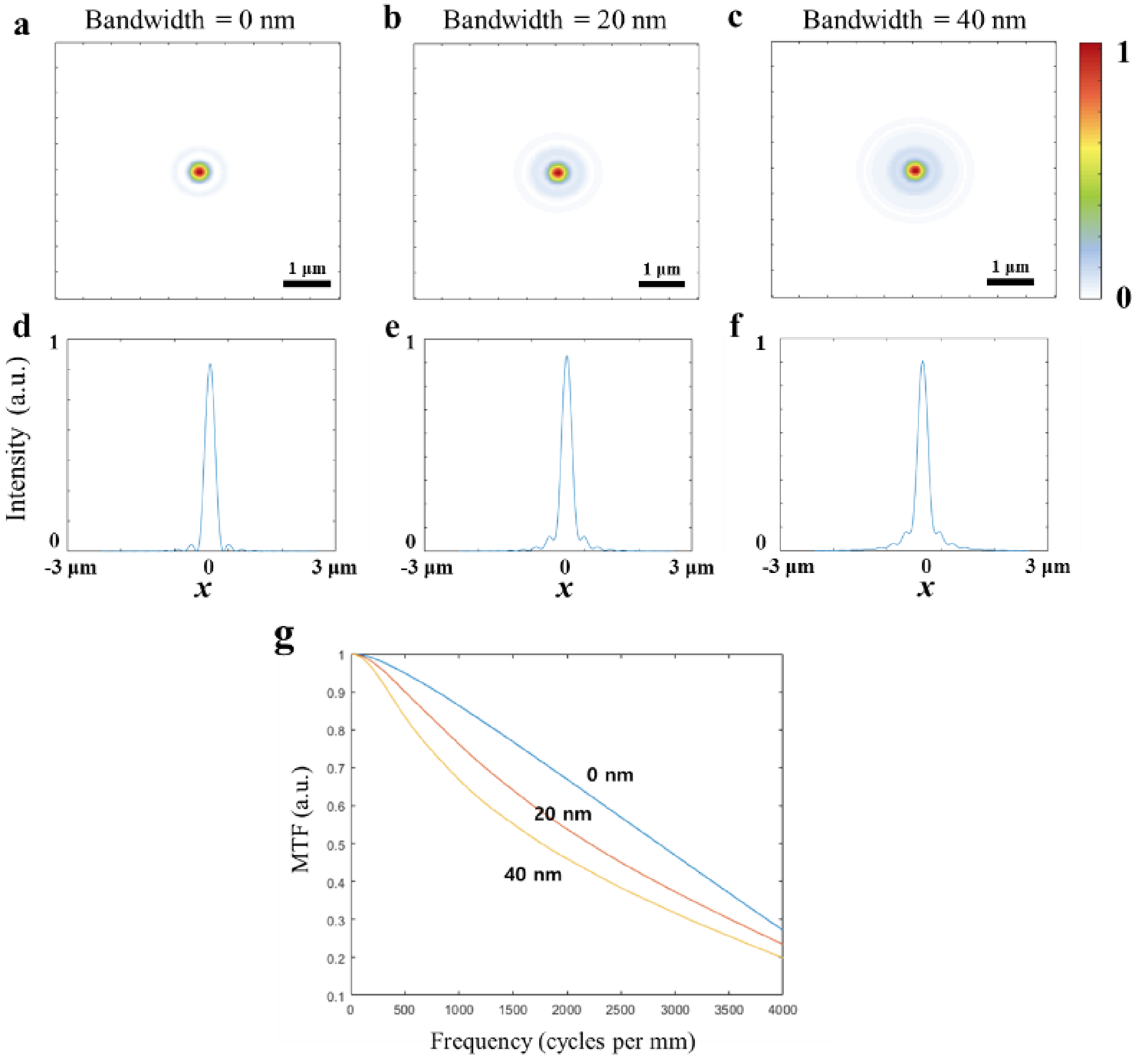

Supplementary Figure 12. MTF analyses according to the source bandwidth (a-c) Field distributions at the focal length, (d-e) the corresponding intensity distributions near the focal point, and (g) MTF curves for several bandwidth conditions when the central wavelength of the incident beam is 532 nm and the numerical aperture is 0.61. Three bandwidths of (a, d) 0 nm (ideal case), (b, e) 20 nm and (c, f) 40 nm are considered.

Supplementary Note 11. Chromatic aberration of the metalens

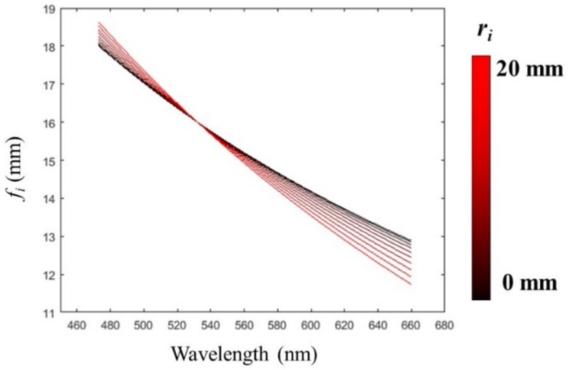

Supplementary Figure 13. Chromatic aberration of the metalens. This figure shows the change of focal lengths in terms of the wavelength and the radius (that is a distance from the center of the metalens)

In this part, the chromatic behavior of the metalens is analyzed. Because the geometric phase is independent of the wavelength of the incident light, the phase profile from the metalens is constant with respect to the wavelength. This results in the chromatic aberration because different phase profiles are required to equalize all focal lengths for different wavelengths. The change in focal length according to the wavelength was well discussed in previous studies, but a more accurate discussion of the variation in focal spot has not been discussed in detail. The relationship of the variation can be determined using the phase matching condition as

$$\phi(r) = \frac{2\pi}{\lambda_d}(f_d - \sqrt{r^2 + f_d^{\,2}}) = \frac{2\pi}{\lambda_i}(f_i - \sqrt{r^2 + f_d^{\,2}}), \tag{7}$$

which means the focal length f_i for the wavelength λ_i of can be directly calculated in terms of the design wavelength λ_{d} and focal length f_{d}. It is notable that the f_i is a function of the radius r, which is the radial distance from the center of the metalens. That means the focal positions from the different r are not constant, resulting to the blurring the focal spot. In other words, this effect makes the chromatic aberration in imaging, so the MTF for the wavelength of 660 nm is quite lower than other wavelengths as we can see in Fig. 2g, agreeing with the results represented in Fig. S13. In conclusion, the metalens has chromatic aberration to imaging quality as well as effective focal length when the metalens is applied to wavelengths other than the design wavelength. This can be corrected through the use of other phase profiles or further development of the metalens such as doublet metalens or achromatic metalens. Using a holographic method to provide virtual information can also be a good candidate to evade this issue.

Supplementary Note 12. Fabrication process using nanoimprint technique

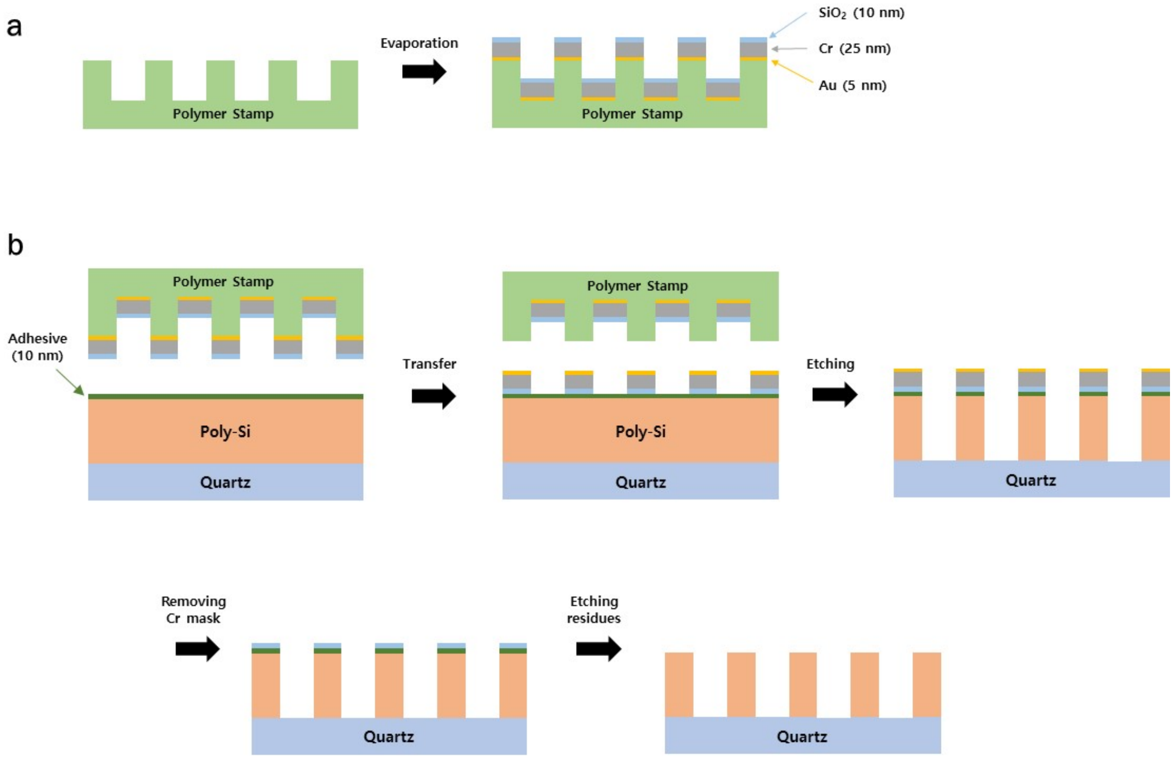

Supplementary Figure 14. Schematics representing the fabrication process of the proposed metasurface based on nanoimprint. There are two steps in overall process. (a) Using a standard electron beam lithography process, a polymer stamp having the metasurface pattern is prepared to be applied to the nanoimprint. Several films are then evaporated using an electron beam evaporator. (b) For the target sample, a quartz wafer with a Poly-Si film and adhesive layer is prepared using LPCVD and spin coating. Using the prepared polymer stamp, the metasurface pattern of the Au, Cr and SiO_2 films is transferred to the wafer. Then, the sample is etched where the Au and Cr patterns are used as a hard mask for etching. After removing Cr mask and other residues by the Cr etchant and further etching process, the sample is finally made.
