# Supplementary material for: Metasurface eyepiece for augmented reality
Source: Nat Commun. 2018 Nov 1;9:4562. doi: 10.1038/s41467-018-07011-5 (PMC6212528; doi:10.1038/s41467-018-07011-5)
Supplement: Supplementary file 3 — Description of Additional Supplementary Files [file 41467_2018_7011_MOESM3_ESM.docx]

**Title:** Supplementary Movie 1

**Description:** This video shows the continuous change of the augmented images in Figure 3d. The first part of the video is for the upper part of Figure 3d showing three rotating cubes, and the second part is for the lower part of Figure 3d which represents a moving shark with an emergency mark. These are repeated several times during the entire play time.
